# Melanin Deposition and Screening of Melanogenesis-Related Differential RNAs and Construction of ceRNA Regulatory Network in Liancheng White Ducks

**DOI:** 10.3390/ani16121891

**Published:** 2026-06-18

**Authors:** Wenli Shi, Li Li, Bangzhe Zhao, Qiannan Cai, Xiaopan Liu, Zhiming Zhu, Linli Zhang, Zhongwei Miao, Qinlou Huang, Nenzhu Zheng, Qingwu Xin

**Affiliations:** 1 Institute of Animal Husbandry and Veterinary Medicine, Fujian Academy of Agricultural Sciences, Fujian Key Laboratory of Animal Genetics and Breeding, Fuzhou 350013, China; swli795@163.com (W.S.); lily102524@163.com (L.L.); 15516113927@163.com (B.Z.); caiqn7@163.com (Q.C.); lxp19940@163.com (X.L.); zzm10203@163.com (Z.Z.); zhll2701010@163.com (L.Z.); 9951044@163.com (Z.M.); hql202@126.com (Q.H.); 2 College of Animal Science, Fujian Agriculture and Forestry University, Fuzhou 350002, China

**Keywords:** Liancheng white duck, melanin, mRNAs, miRNAs, lncRNAs

## Abstract

Liancheng white ducks have a distinctive “white feathers, black beak, and green feet” phenotype, making them a useful model for studying pigmentation traits in waterfowl. The previous study found that the F1 generation of Liancheng white ducks crossed with white-feathered ducks and hemp-feathered ducks were all gray-black in color. This indicates the specificity and complexity of melanin deposition in Liancheng white ducks, which makes the selection and breeding of pigment traits through phenotyping difficult. The aim of this study was to investigate the candidate transcriptomic regulatory signals of melanogenesis in Liancheng white ducks. Skin, mouth skin, foot skin, liver, and muscle samples were collected from 130-day-old Liancheng white ducks. Morphological differences were observed via histological analysis, and extraction-based pigment levels were determined. The results showed that melanin granules were clearly observed in tissues other than the liver and were distributed mainly in the basal layer of the epidermis and around feather follicles; the pigment values in the tissues decreased in the order mouth skin > liver > foot skin > muscle and skin. However, the relatively high liver value should be interpreted cautiously because obvious melanin granule deposition was not observed histologically. Whole-transcriptome sequencing was performed on mouth skin and skin samples. In total, 3074 differentially expressed genes (DEGs) were screened; upregulated genes associated with melanogenesis included melanocyte inducing transcription factor (MITF) and tyrosinase (TYR); downregulated genes included agouti signaling protein (ASIP) and adenylate cyclase 2 (ADCY2). Eighteen differentially expressed microRNAs (DEmiRNAs) were identified. Based on target prediction and pathway enrichment analysis, novel_290 and apl-miR-11588-3p were identified as candidate miRNAs potentially associated with melanogenesis-related pathways, and their predicted target genes included phosphatidylinositol 3-kinase (PI3K) and Janus kinase 1 (JAK1). Additionally, 364 differentially expressed long noncoding RNAs (DElncRNAs) were identified; TCONS_00063335 and TCONS_00019814 were identified as candidate lncRNAs potentially associated with melanogenesis-related genes, including TYR and TYRP1. A putative ceRNA network was constructed based on the predicted miRNA–mRNA and miRNA–lncRNA relationships, and ENSAPLT00000025522–apl-miR-11588-3p–MAPK8IP3 was identified as a candidate network relationship associated with MAPK-related pigmentation pathways. However, because this relationship was inferred mainly from bioinformatic prediction and expression association analysis, further functional validation is required to confirm whether it contributes to melanogenesis regulation. These findings provide candidate transcriptomic and noncoding RNA information for the further investigation of tissue-specific pigmentation in Liancheng white ducks.

## Simple Summary

This study explored how a unique duck breed, Liancheng white ducks, controls the production of melanin—the pigment responsible for coloration. This study examined skin, mouth skin, foot skin, liver, and muscle tissues from ducks. We found melanin in most tissues (except the liver), concentrated in the skin’s deepest layer and around hair follicles. The mouth skin showed the highest extraction-based pigment value, whereas the liver showed a relatively high extraction-based pigment signal despite the absence of obvious melanin granules in histological sections. These findings suggest that liver pigment signals require further melanin-specific validation. By analyzing genetic activity, we identified key genes that may regulate pigment production. For example, certain genes linked to pigment creation were more active, while others that limited pigmentation were less active. We also discovered microRNAs (miRNAs) and long noncoding RNAs (lncRNAs) that might work together to control these genes. Lab tests confirmed the accuracy of these findings. This research helps explain how these ducks manage their coloration and could inspire broader studies on animal pigmentation.

## 1. Introduction

The Liancheng white duck, also called the white-feathered duck, is a valuable and rare local waterfowl germplasm resource in China and a small white shelduck variety with provincial characteristics. It has white feathers, a black beak, and green feet, making it a useful model for studying pigmentation in domestic waterfowl [1]. Previous studies [1,2] showed that when crossed with other white-feathered domestic duck breeds, all F1 offspring are gray or black, different from other white-feathered breeds. The specificity and complexity of melanin deposition in Liancheng white ducks make it hard to breed based on phenotype alone. Thus, analyzing the melanin formation mechanism at the molecular level can offer insights for breeding Liancheng white ducks.

Coloration is not only an animal phenotypic marker but also an important breeding indicator. Duck feather, beak, and foot (including shank and interdigital webbing) colors can vary, and coloration is important for duck husbandry. Due to market preference for specific duck coloration patterns, specific duck strains should be developed to enhance the market competitiveness of duck products. Besides embellishing appearance, beak and foot colors have become important consumer choice factors in traditional consumption, highlighting their importance in recent breeding research and production. Duck beak and foot colors, as qualitative traits, should be studied to explore genetic patterns and scientifically preserve local breed characteristics, promoting duck breeding, production, development, and utilization.

Melanin is a biological macromolecular pigment formed from phenolic or indole compounds via complex reactions. It is widely distributed in animals, plants, and microorganisms [3]. In animals, melanocytes mainly produce eumelanin and pheomelanin. Eumelanin is responsible for black and brown pigmentation [4]. Pheomelanin contributes to red, yellow, and reddish-brown coloration [5]. Melanin synthesis occurs in melanosomes and involves melanocyte development, migration, melanosome transport, and melanin deposition, all of which are regulated by multiple pigmentation-related genes and signaling pathways [6,7]. Among them, tyrosinase is a key rate-limiting enzyme in melanin synthesis, and together with tyrosinase-related protein 1 (TYRP1) and dopachrome tautomerase (DCT) play an important role in eumelanin production [8]. In livestock and poultry, pigmentation studies have mainly focused on coding genes such as MITF, TYR, ASIP, melanocortin 1 receptor (MC1R), and endothelin 3 (EDN3), as well as their sequence variation and functional roles. For example, Zhang et al. [9] identified single nucleotide polymorphism (SNP) in the coding region of MC1R in a local chicken breed, and Yu et al. [10] reported that two glutathione (GSH) metabolism-related genes (CHAC1 and GPX3) were involved in melanin production in black-bone chicken feathers.

Meanwhile, ceRNAs bind to miRNAs via microRNA response elements (MREs) to disable them, regulating gene expression. As high-throughput sequencing technology develops, more novel miRNAs and lncRNAs are discovered, leading to more studies on the biological functions. However, most existing studies have focused on individual genes or limited regulatory pathways, and the integrated regulatory roles of mRNAs, lncRNAs, and miRNAs in tissue-specific melanin deposition remain insufficiently understood, especially in Liancheng white ducks. Therefore, investigating the transcriptomic and non-coding RNA regulatory mechanisms underlying melanin deposition in this breed may provide new insight into the molecular basis of pigmentation traits in ducks.

Therefore, this study used Liancheng white duck, a local breed in Fujian Province, China, as the research object. Through histological analysis and the measurement of tissue melanin content, the deposition locations and amount of melanin in Liancheng white duck tissues were clarified. Whole-transcriptome sequencing was performed on the mouth skin and skin tissue to identify differentially expressed mRNAs, miRNAs, and lncRNAs in the tissues and to construct an lncRNA-miRNA-mRNA regulatory network. This study not only lays the foundation for understanding the specific mechanism of melanogenesis in Liancheng white duck tissues, but also provides a reference for the selection and breeding of phenotypic traits like black beak and green feet in Liancheng white ducks.

## 2. Materials and Methods

### 2.1. Experimental Animals and Materials

Twenty-four healthy 130-day-old Liancheng white duck females, with an average body weight of approximately 1.35 kg, were provided by the Poultry Germplasm Innovation and Facility Cage-Rearing Demonstration Base of the Institute of Animal Husbandry and Veterinary Medicine, Fujian Academy of Agricultural Sciences. All ducks were raised under standardized husbandry conditions, with consistent nutritional management and environmental control throughout the rearing period. The diet composition and nutritional levels are shown in Table S1. The 24 ducks were allocated into four biological replicate groups, with six ducks in each group. After 12 h of fasting, the ducks were euthanized by severing the carotid artery immediately after electrocution in a water bath. In each group, one duck was randomly selected for histological analysis. The surfaces of the beak and feet were disinfected by wiping with 75% ethanol and allowed to air-dry. Mouth skin and foot skin tissues were then rapidly excised from the target sites, and skin and liver tissues were subsequently dissected. All tissue samples were individually fixed in 4% paraformaldehyde for subsequent paraffin section preparation (*n* = 4). From the remaining five ducks in each group, the skin, mouth skin, foot skin, liver, and muscle were collected. Equal amounts of the same tissue from the five ducks within each group were pooled to generate one mixed sample per tissue type. Two pooled samples were prepared for each tissue type. One pooled sample was stored at −20 °C for melanin content determination, while the other pooled sample was immediately frozen in liquid nitrogen and then stored at −80 °C for subsequent RNA extraction. Thus, four pooled biological replicates were obtained for each tissue type.

### 2.2. Section Preparation

The tissues were removed from 4% paraformaldehyde fixative and rinsed with distilled water to remove excess formaldehyde. The tissues were trimmed flat with a scalpel at the target site and then tissues were put into dehydration boxes and numbered, dehydrated, cleared, wax dipped, embedded, sectioned, spread, and baked. The paraffin sections were then dewaxed and rehydrated, the tissue samples were stained using hematoxylin and eosin (HE) and toluidine blue (TB) staining techniques and sealed, and the melanin deposition in each tissue sample was observed under a microscope (Nikon, ECLIPSE Ni-L, Tokyo, Japan).

### 2.3. Melanin Content Testing

The assay was performed using an acid-hydrolysis-based method, modified from a previously published melanin extraction and purification protocol [11,12].

The tissue samples were dried at 100 °C and ground to powder. A total of 10 g was weighed, concentrated hydrochloric acid was added, and the mixture was soaked until a homogeneous suspension formed. Methanol was added, and the mixture was centrifuged at 12,000 r/min for 20 min, after which the supernatant was discarded. Approximately 200 mL (1:4) of hydrochloric acid–ethanol mixture was added, and the mixture was placed in a water bath at 80 °C for 24 h and then centrifuged for 20 min. The supernatant was discarded, and acetone and petroleum ether were added to wash the retained solids. After each wash, the mixture was centrifuged for 10 min, the supernatant was discarded, an appropriate amount of anhydrous ethanol was added, the ethanol suspension was poured into a weighing dish containing sea sand, the dish was placed in a 70 °C water bath to evaporate, and the dish was baked in a vacuum electrically heated thermostatic drying oven until a constant weight (80 °C, −0.09 MPa) was reached. The formula is as follows:

(1) $$X,\%=\frac{M_{2}-M_{1}}{M}\times 100$$

where X is the melanin content of the sample (dry basis), in %; M is the mass of the sample after drying and grinding, in g; M_1_ is the mass of the weighing dish containing sea sand without melanin, in g; and M_2_ is the mass of the weighing dish containing sea sand with melanin, in g.

### 2.4. Library Construction, Library Testing, and Sequencing

After the determination of tissue melanin content, the frozen pooled samples corresponding to the tissues with the highest and lowest melanin contents were selected for whole-transcriptome sequencing analysis. Total RNA was extracted from the samples using TRIzol reagent (Invitrogen, Carlsbad, CA, USA), followed by strict quality control of the RNA samples. The tested samples were used to establish RNA-Seq libraries and miRNA-Seq libraries, reverse transcription and amplification were used to create cDNA libraries, and Qubit 2.0 was then used for preliminary quantification. The library insert sizes were determined using a high-sensitivity Agilent 2100 (Agilent Technologies, Santa Clara, CA, USA), and after the insert size met the expected values, the libraries were accurately quantified (>2 nM) via the qPCR method. After passing the library inspection, the library was sequenced using the Illumina HiSeq 2000 (Illumina, Inc., San Diego, CA, USA) sequencing platform with single-end 50 bp (SE50) reads for miRNAs and paired-end 150 bp (PE150) reads for mRNAs and lncRNAs by Novogene Co., Ltd. (Beijing, China).

### 2.5. Bioinformatics Analysis

FastQC software (v0.11.9) was utilized to assess the quality of the raw data, low-quality reads and adapter sequences were removed using fastp, reads with ambiguous or low-confidence alignments were excluded in subsequent analyses, and the clean data were aligned to the reference genome (BGI_duck_1.0, GCA_000355885.1). Fragments were spliced into transcripts and quantified using StringTie (v1.3.3) software based on the results of the comparison to the genome. The transcripts obtained from the splicing of each sample were merged using Cufflink software (v2.1.1) and compared with known databases, transcripts present in databases were filtered out, and potential coding predictions were carried out on the filtered new transcripts to obtain novel lncRNAs and novel mRNAs, which were analyzed by Cluster (v3.2.4) clustering. Then, the expression levels were quantified, and differential expression significance analysis was performed using edgeR (v3.24.3) software, with adjusted *p*-value < 0.05 and |log_2_ (Fold Change)| ≥ 1 as the significance criterion. Finally, lncRNA target genes were predicted via the positional relationship (colocation) and correlation in expression (coexpression) between lncRNAs and protein-encoding genes.

Sequencing raw data filtering was performed as above, followed by small RNA length screening (18–35 nt), and the screened sRNAs were then aligned to the reference genome (BGI_duck_1.0, GCA_000355885.1). Next, known miRNA analysis (miRDeep2, v0.0.5) [13], novel miRNA prediction (miREvo, v1.1) [14], miRNA expression normalization, and differential expression analysis (DEG Seq, v1.38.0) were performed [15]. The R software SAM (v3.0) was used to screen the DEmiRNAs and perform cluster analysis. miRanda (v3.3a), PITA, and RNAhybrid (v2.0) were used to predict miRNA target genes to determine the correspondence between miRNAs and target genes [16].

Finally, differentially expressed genes and the candidate target genes of novel lncRNAs and novel miRNAs were analyzed via Gene Ontology (v2.12, GO, http://www.geneontology.org/) and Kyoto Encyclopedia of Genes and Genomes (KEGG) functional annotation analysis.

### 2.6. qRT-PCR Validation

Quantitative reverse transcription polymerase chain reaction (qRT-PCR) primers were designed using Primer Premier (v6.0) and Beacon designer (v7.8) and then synthesized by Sangon Biotech Co., Ltd. (Shanghai, China). The primer sequences are shown in Tables S2 and S3. The internal reference genes used were glyceraldehyde-3-phosphate dehydrogenase (GAPDH) and U6. The reaction system (total volume, 20 μL): 10 μL Power SYBR Green Master Mix, 0.5 μL each of forward and reverse primers (10 μM), 1 μL cDNA template, and 8 μL sterile distilled water (SDW). Amplification conditions: initial denaturation at 95 °C for 1 min; followed by 40 cycles of 95 °C for 15 s and 63 °C for 25 s (annealing and extension). Each sample was repeated three times to ensure the accuracy and repeatability of the test results. The relative expression levels of genes were calculated and analyzed via the 2^−ΔΔCt^ method.

### 2.7. Statistical Analysis

The data of melanin content and qRT-PCR relative expression data (2^−ΔΔCt^ values) were analyzed using SPSS (v27.0.). Data normality and homogeneity of variance were assessed using the Shapiro–Wilk test and Levene’s test, respectively. When the assumptions of normality and homogeneity of variance were met, one-way analysis of variance (ANOVA) was used to identify significant differences among groups, followed by Duncan’s multiple range test for post hoc comparisons. Duncan’s test was used to assign statistical groupings among multiple tissues and to maintain consistency with the letter-based significance annotations in the figures. *p* < 0.05 (*) was considered a significant difference, and *p* < 0.01 (**) was considered an extremely significant difference. The data are presented as the means ± standard deviations (SDs).

For transcriptome analyses, differential expression analysis of mRNAs, lncRNAs, and miRNAs was performed using the corresponding bioinformatics tools described above. To control for multiple testing, *p*-values were adjusted using the Benjamini–Hochberg false discovery rate (FDR) method. Differentially expressed RNAs were identified using an adjusted *p*-value < 0.05 and |log_2_ (Fold Change)| ≥ 1 as thresholds. For GO and KEGG enrichment analyses, multiple-testing correction was also performed using the Benjamini–Hochberg method, and pathways or terms with adjusted *p* < 0.05 were considered significantly enriched. Pathways discussed without meeting the adjusted significance threshold were interpreted as candidate pathways for further investigation.

## 3. Results

### 3.1. Histological Analysis of Melanin Deposition in Liancheng White Ducks

**Figure 1:**
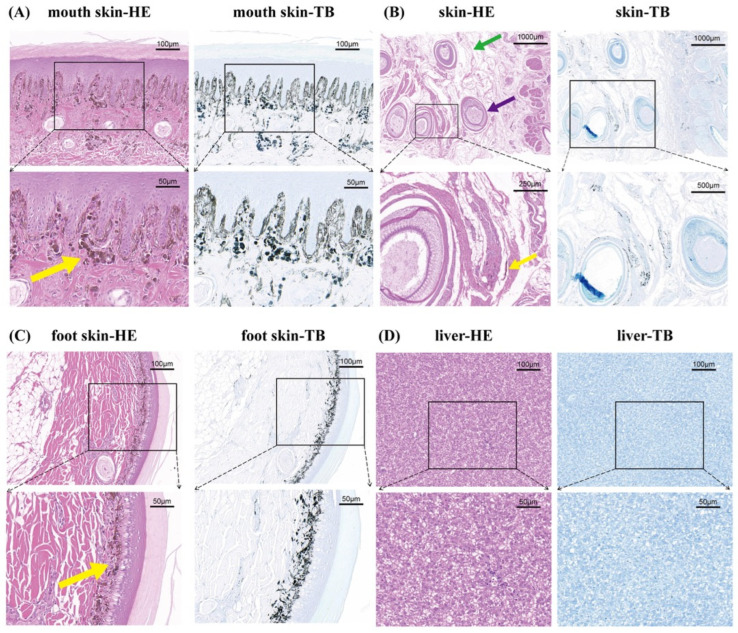
Histological observation of melanin deposition in different tissues of Liancheng white ducks. Representative paraffin sections of mouth skin, skin, foot skin, and liver were stained with HE and TB. HE staining turned the nuclei blue and other tissues pink, allowing for clear observation of tissue structures and distinct visualization of melanin granules. Toluidine blue staining rendered collagen fibers and the epidermal stratum corneum vividly blue, facilitating the observation of melanin granules. The upper images show the low-magnification views, and the lower images show the enlarged regions corresponding to the black boxes in the upper images. Scale bars are indicated in the upper-right corner of each micrograph. (**A**) HE and TB staining of mouth skin showed a large amount of melanin in the basal layer of the epidermis. (**B**) HE and TB staining of skin. Melanin granules were rare and observed only around a few hair follicles and within interstitial spaces. (**C**) HE and TB staining of foot skin showed a large amount of melanin in the basal layer of the epidermis. (**D**) HE and TB staining of liver showed no obvious melanin granule deposition. The yellow arrows indicate melanin; the green arrows indicate collagen fibers; the purple arrows indicate hair follicles. Black boxes indicate the regions selected for higher-magnification observation.

To observe the tissue structure more clearly, the tissue sections were sectioned longitudinally in this study. As shown in Figure 1, the sections completely displayed the epidermis, dermis, and subcutaneous tissue of the mouth skin, skin, and foot skin; the morphology of the melanocytes was vacuolar, and there were many melanin granules in and around the cells.

Mouth skin sections (Figure 1A) contained a large amount of melanin in the basal layer of the epidermis, and the collagen fibers in the dermis were neatly arranged with a large amount of melanin.

In the skin section (Figure 1B), the collagen fibers of the dermis were disordered, and rare melanin granules were observed around a few hair follicles and in between collagenous fibrous tissues.

Foot skin sections (Figure 1C) revealed that the collagen fibers in the dermis were neatly arranged, and a large amount of melanin could be seen in the basal layer of the epidermis. In the field of view (Figure 1D), the liver cells had round nuclei, were abundant and neatly arranged, but no obvious melanin was observed in the tissues. In summary, melanin granules can be clearly observed in skin but not the liver, and melanin granules are distributed mainly in the basal layer of the epidermis and around hair follicles.

### 3.2. Melanin Deposition in Different Tissues of the Liancheng White Duck

**Figure 2:**
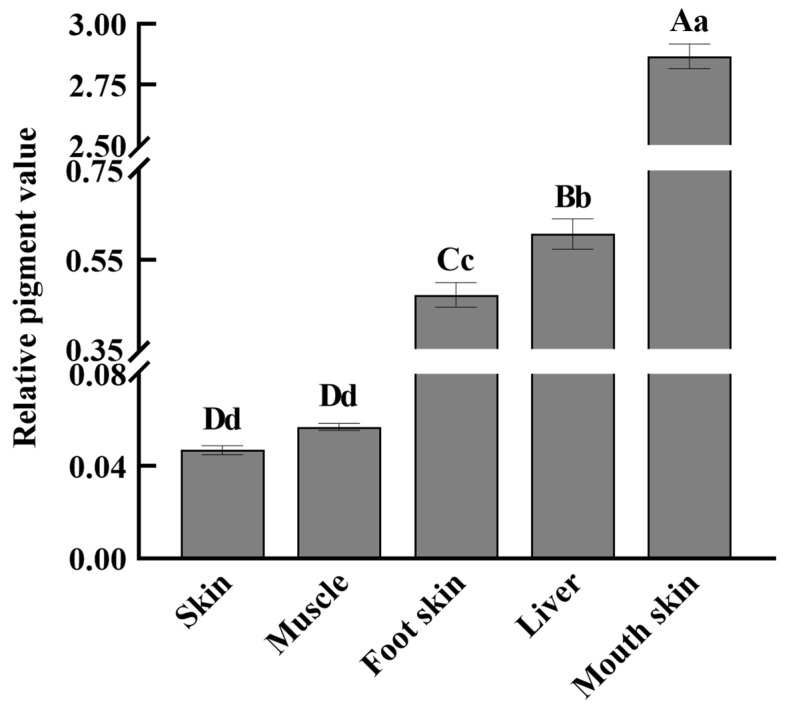
Pigment values in different tissues of Liancheng white ducks. The X-axis represents different tissue types, including skin, muscle, foot skin, liver, and mouth skin; the Y-axis represents the data of relative pigment values of melanin. The Y-axis was broken to allow for visualization of both low- and high-abundance tissues within the same plot. The data are presented as the means ± SD, with error bars representing the standard deviation. Each tissue type contained four pooled biological replicates (*n* = 4), and each pooled replicate was generated from equal amounts of the same tissue collected from five ducks within one biological replicate group. Mouth skin showed the highest pigment values, followed by liver and foot skin, whereas skin and muscle showed the lowest pigment values. Whereas different uppercase letters indicate significant differences among tissues (*p* < 0.05), different lowercase letters indicate extremely significant differences (*p* < 0.01), and the same letters indicate no significant difference (*p* > 0.05).

Figure 2 shows that the Liancheng white duck mouth skin tissue had the highest pigment value, which was significantly greater than that of the other tissues (*p* < 0.01), and the skin and muscle tissues presented the lowest pigment value, which was significantly lower than that of the other tissues (*p* < 0.01). The order of tissues with the highest to lowest pigment value was mouth skin > liver > foot skin > muscle and skin.

### 3.3. Analysis of DEGs Between Mouth Skin and Skin Tissues

**Figure 3:**
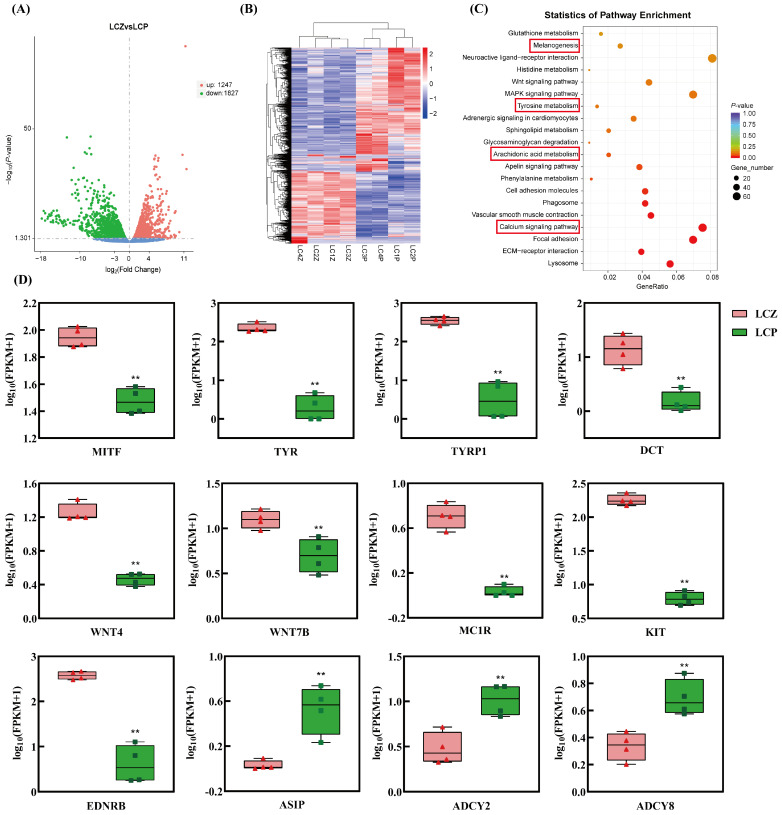
Differentially expressed gene analysis between mouth skin and skin of Liancheng white ducks. LCZ represents mouth skin, LCP represents skin. Four pooled biological replicates were included in each group (*n* = 4). (**A**) Volcano plot showing DEGs between LCZ and LCP. Red dots represent upregulated genes, green dots represent downregulated genes, and blue dots represent genes without significant differential expression. The X-axis indicates log_2_ (Fold Change), and the Y-axis indicates −log_10_ (*p*-value). The horizontal dashed line indicates the significance threshold of *p*-value = 0.05, and the vertical dashed line indicates log_2_(Fold Change) = 0. (**B**) Hierarchical clustering heatmap of DEGs between LCZ and LCP. Rows represent genes, and columns represent samples. Red indicates higher expression levels, while blue indicates lower expression levels. (**C**) KEGG pathway enrichment analysis of DEGs. The X-axis represents the GeneRatio, calculated as the number of DEGs enriched in a specific pathway divided by the total number of DEGs. Dot size indicates the number of enriched genes, and dot color indicates the enrichment *p*-value. Red boxes highlight pigmentation-related pathways. (**D**) Expression levels of selected pigmentation-related DEGs in LCZ and LCP. The Y-axis represents log_10_ (FPKM + 1). Horizontal lines represent the median and the small triangular or square symbols represent biological replicates. ** indicates *p* < 0.01.

A total of 3074 DEGs were screened to compare the tissues with the greatest differences in melanin content, mouth skin, and skin tissues of Liancheng white ducks, and in the mouth skin tissue, 1247 DEGs were upregulated, and 1827 DEGs were downregulated (Figure 3A).

Hierarchical clustering analysis was performed based on the fragments per kilobase of transcript per million fragments mapped (FPKM) values of the DEGs in mouth skin and skin tissues, and the heatmap visually depicted the similarity in the expression levels of genes among different samples in the same tissue and the expression patterns between mouth skin and skin tissues. These findings indicate that the sample selection was reasonable and could be used for subsequent analysis, as shown in Figure 3B.

KEGG pathway enrichment analysis was performed on the DEGs using clusterProfiler software (v4.4.4) (Figure 3C). The results (Table 1) revealed significant differences in the lysosome, ECM–receptor interaction, focal adhesion pathway, calcium signaling pathway, arachidonic acid metabolism, tyrosine metabolism, melanogenesis, and other pathways (*p* < 0.05). The upregulated genes screened from this analysis included MITF, TYR, TYRP1, DCT, Wnt family member 4 (WNT4), Wnt family member 7B (WNT7B), MC1R, and receptor tyrosine kinase (KIT), and endothelin receptor type B (EDNRB); the downregulated genes included ASIP, ADCY2, and adenylate cyclase 8 (ADCY8) (Figure 3D).

### 3.4. Analysis of DEmiRNAs Between Mouth Skin and Skin

LC3Z was excluded after sequencing QC because its library quality did not meet the inclusion criteria. The remaining three biological replicates in this group passed QC and were retained for downstream analyses, which satisfies the biological replicate requirement for our transcriptome analysis.

**Figure 4:**
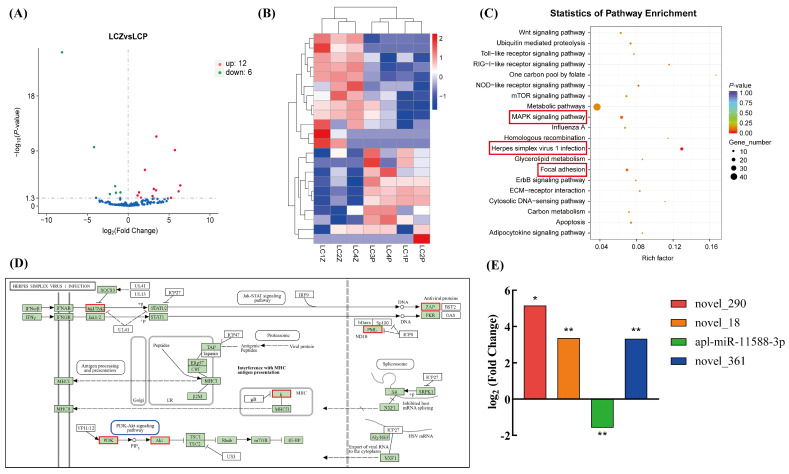
Differentially expressed miRNA analysis between mouth skin and skin of Liancheng white ducks. LCZ represents mouth skin, and LCP represents skin. After sequencing quality control, LC3Z was excluded because its library quality did not meet the inclusion criteria; therefore, three biological replicates were retained in the LCZ group, and four biological replicates were retained in the LCP group for downstream miRNA analysis. (**A**) Volcano plot of DEmiRNAs between LCZ and LCP. Red dots indicate upregulated miRNAs, green dots indicate downregulated miRNAs, and blue dots indicate miRNAs without significant differential expression. The X-axis indicates log_2_ (Fold Change), and the Y-axis indicates −log_10_ (*p*-value). The horizontal dashed line indicates the significance threshold of *p*-value = 0.05, and the vertical dashed line indicates log_2_ (Fold Change) = 0. (**B**) Hierarchical clustering heatmap of DEmiRNAs. Rows represent DEmiRNAs, and columns represent individual samples. Red indicates relatively high expression, whereas blue indicates relatively low expression. (**C**) KEGG pathway enrichment analysis of predicted target genes of the DEmiRNAs. The X-axis represents the Rich factor. Dot size indicates the number of target genes enriched in each pathway, and dot color indicates the enrichment *p*-value. Red boxes highlight pigmentation-related pathways. (**D**) KEGG pathway map of the herpes simplex virus 1 infection pathway. Red boxes indicate upregulated genes enriched in this pathway and associated with the predicted target genes of DEmiRNAs. In the pathway map, solid arrows indicate activation, promotion, or the direction of signal transduction; blunt-ended lines indicate inhibition or suppression; and dashed arrows indicate indirect relationships, translocation, or process flow according to the KEGG pathway notation. (**E**) The log_2_ (Fold Change) values of representative DEmiRNAs, including novel_290, novel_18, apl-miR-11588-3p, and novel_361. Positive values indicate upregulation in mouth skin, whereas negative values indicate downregulation. * indicates *p* < 0.05, and ** indicates *p* < 0.01.

A total of 18 DEmiRNAs were screened (Figure 4A), and in the mouth skin tissue, 12 DEmiRNAs were upregulated, and 6 DEmiRNAs were downregulated. A hierarchical cluster analysis was performed (Figure 4B).

The target gene prediction results revealed that for 18 DEmiRNAs, a total of 918 target genes were predicted. KEGG pathway analysis was performed on related target genes to further understand the function of these miRNAs in the control of melanin deposition (Figure 4C). The KEGG annotation data for the target genes of the DEmiRNAs revealed that the herpes simplex virus 1 infection pathway, MAPK signaling pathway, focal adhesion pathway, NOD-like receptor signaling pathway, RIG-I-like receptor signaling pathway, and other pathways were significantly enriched (*p* < 0.05).

The herpes simplex virus 1 infection pathway (Figure 4D) included the target genes PI3K and JAK1, and the corresponding miRNAs included novel_144 and novel_290 for the former and novel_18 and novel_54 for the latter.

The MAPK signaling pathway included the target genes mitogen-activated protein kinase 8 interacting protein 3 (MAPK8IP3), and the corresponding miRNAs were apl-miR-11588-3p and novel_144.

The target gene integrin linked kinase (ILK) was included in the focal adhesion, and the corresponding miRNAs were novel_171, novel_235, and novel_361.

Based on pathway-guided filtering of the KEGG-enriched results and miRNA-target gene mapping, four representative DEmiRNAs (novel_290, novel_18, apl-miR-11588-3p, and novel_361) were highlighted as candidates potentially involved in melanin deposition (Figure 4E).

### 3.5. Screening of DElncRNAs Between Mouth Skin and Skin

**Figure 5:**
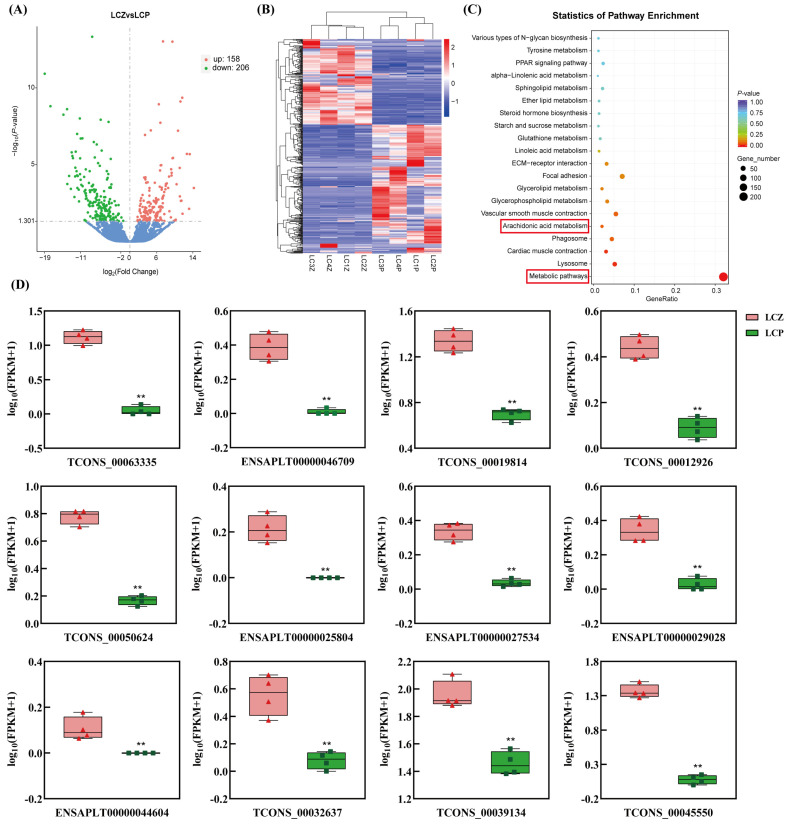
Differentially expressed lncRNA analysis between mouth skin and skin of Liancheng white ducks. LCZ represents mouth skin, and LCP represents skin. Four pooled biological replicates were included in each group (*n* = 4). (**A**) Volcano plot of DElncRNAs between LCZ and LCP. Red dots indicate upregulated lncRNAs, green dots indicate downregulated lncRNAs, and blue dots indicate lncRNAs without significant differential expression. The X-axis indicates log_2_ (Fold Change), and the Y-axis indicates −log_10_ (*p*-value). The horizontal dashed line indicates the significance threshold of *p*-value = 0.05, and the vertical dashed line indicates log_2_ (Fold Change) = 0. (**B**) Hierarchical clustering heatmap of DElncRNAs. Rows represent lncRNAs, and columns represent individual samples. Red indicates relatively high expression levels, whereas blue indicates relatively low expression levels. (**C**) KEGG pathway enrichment analysis of the predicted target genes of DElncRNAs. The X-axis represents the GeneRatio, calculated as the number of target genes enriched in a specific pathway divided by the total number of target genes. Dot size indicates the number of enriched genes, and dot color indicates the enrichment *p*-value. Red boxes highlight pigmentation-related pathways. (**D**) Expression levels of representative DElncRNAs in LCZ and LCP. The Y-axis represents log_10_ (FPKM + 1). Horizontal lines represent the median and the small triangular or square symbols represent biological replicates. ** indicates *p* < 0.01.

A total of 364 DElncRNAs were screened (Figure 5A), and in the mouth skin tissue, 158 DElncRNAs were upregulated, and 206 DElncRNAs were downregulated; hierarchical clustering analysis was subsequently performed (Figure 5B).

LncRNAs can control gene expression through cis-regulatory and trans-regulatory mechanisms. KEGG pathway analysis was performed on relevant target genes (Figure 5C). The KEGG annotation data revealed that metabolic pathways, lysosome, cardiac muscle contraction, the arachidonic acid metabolism pathway, and other pathways were significantly enriched (*p* < 0.05).

The metabolic pathways included the target genes TYR and TYRP1, and the corresponding DElncRNAs were TCONS_00063335, ENSAPLT00000046709, TCONS_00019814, and TCONS_00012926.

The arachidonic acid metabolism pathway included the target genes carbonyl reductase 1 (CBR1), hematopoietic prostaglandin D synthase (HPGDS), and glutathione peroxidase 2 (GPX2), and the corresponding DElncRNAs were TCONS_00063335, TCONS_00019814, and TCONS_00012926; TCONS_00019814 and TCONS_00012926; and TCONS_ 00050624, respectively (Figure 5D).

### 3.6. lncRNA–miRNA–mRNA Regulatory Network

**Figure 6:**
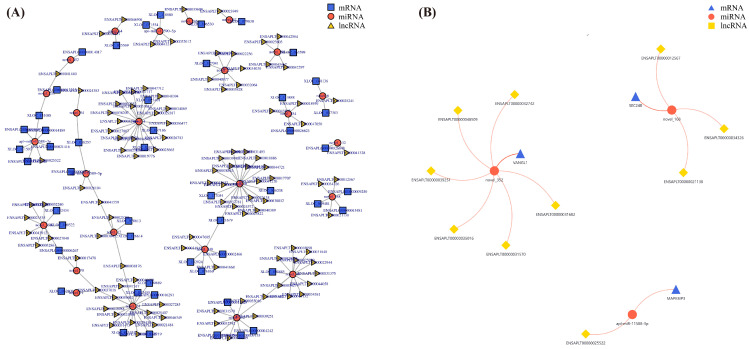
ceRNA regulatory network analysis between mouth skin and skin of Liancheng white ducks. The ceRNA network was constructed using differentially expressed mRNAs, lncRNAs, and miRNAs based on predicted miRNA–mRNA and miRNA–lncRNA regulatory relationships. The network was visualized using Cytoscape software. (**A**) ceRNA regulatory network constructed from all screened mRNA–miRNA–lncRNA interaction pairs. Different node shapes and colors represent different RNA types: blue nodes represent mRNA, red nodes represent miRNA, and yellow nodes represent lncRNA. Lines indicate predicted regulatory relationships among mRNAs, miRNAs, and lncRNAs. (**B**) Selected ceRNA relationship pairs associated with enriched pathways related to melanin regulation. Different node shapes and colors represent different RNA types: blue nodes represent mRNA, red nodes represent miRNA, and yellow nodes represent lncRNA.

In the miRNA–lncRNA and miRNA–mRNA regulatory networks, lncRNAs and mRNAs predicted to be regulated by the same miRNA were screened, and the putative interaction network was generated using Cytoscape software (v3.9.1) (Figure 6A). KEGG pathway enrichment analysis of the ceRNA regulatory network revealed that the Wnt signaling pathway and protein processing in the endoplasmic reticulum were significantly enriched (*p* < 0.05) (Figure S1). Although the MAPK signaling pathway was not significantly enriched (*p* > 0.05), it is known to be associated with melanin production and was therefore retained as a candidate pathway for further analysis. The MAPK signaling pathway included MAPK8IP3, and the predicted relationship pair ENSAPLT00000025522–apl-miR-11588-3p–MAPK8IP3 was found. The Wnt signaling pathway included VANGL planar cell polarity protein 1 (VANGL1), and six putative ceRNA relationship pairs were identified, including ENSAPLT00000031570–novel_352–VANGL1. Protein processing in the endoplasmic reticulum included SEC24 homolog B (SEC24B), and three putative ceRNA relationship pairs were identified, including ENSAPLT00000012567–novel_168–SEC24B (Figure 6B). These lncRNA–miRNA–mRNA relationships were inferred from bioinformatic prediction and expression association analyses and should be regarded as candidate regulatory interactions requiring further experimental validation.

### 3.7. Validation of Sequencing Results by qRT-PCR

**Figure 7:**
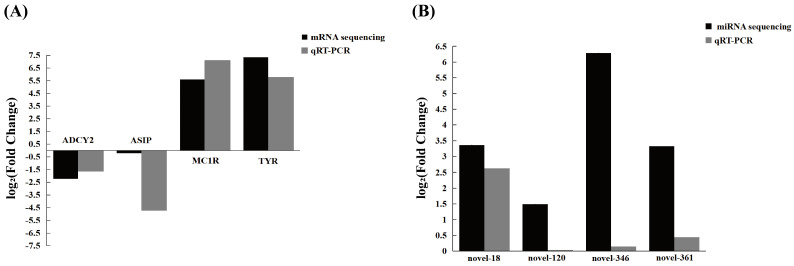
qRT-PCR validation of the RNA-Seq results. qRT-PCR was performed to validate the expression trends of differentially expressed mRNAs and miRNAs between mouth skin and skin of Liancheng white ducks. (**A**) Verification results of DEGs. (**B**) Verification results of DEmiRNAs. Black bars represent fold changes obtained from sequencing data, and gray bars represent fold changes obtained from qRT-PCR. The Y-axis represents log_2_ (Fold Change). Positive values indicate upregulation in mouth skin compared with skin, whereas negative values indicate downregulation. GAPDH was used as the internal reference gene for mRNA validation, and U6 was used as the internal reference for miRNA validation. The generally consistent expression trends between sequencing and qRT-PCR results support the reliability of the transcriptome sequencing data.

To verify the reliability of the high-throughput sequencing results, 2 upregulated genes, 2 downregulated genes, and 4 upregulated miRNAs were selected, and the relative expression levels in the mouth skin and skin tissues were determined via qRT-PCR. The results (Figure 7) show that the expression trends of selected genes and miRNAs are generally consistent with the sequencing data, supporting the observed differential expression patterns.

## 4. Discussion

### 4.1. Distribution and Content of Melanin in Liancheng White Duck Tissues

The distribution and relative abundance of melanin are closely associated with pigmentation traits in animals. Therefore, determining the localization and tissue-specific pigment levels is an important first step for understanding pigmentation in Liancheng white ducks.

In this study, HE staining and TB staining methods were used to analyze the morphology and melanin distribution in the skin, mouth skin, foot skin, and liver tissues of Liancheng white ducks. Compared with previous histological reports in other animal models [17,18,19], our results similarly showed that pigment granules were most prominent in tissues with visible dark phenotypes. Specifically, the number of melanin granules in the mouth skin and foot skin tissues of Liancheng white duck was significantly greater than that in the other tissues, and the melanin granules were obviously distributed in the basal layer of the epidermis, which is consistent with the typical appearance characteristics of “black beak and green feet”. In skin tissues, melanin was distributed mainly around feather follicles, but the amount of melanin deposited was far lower than that deposited on the mouth skin. Nganvongpanit et al. [17] studied the distribution of melanin in 33 organs of Thai black-bone chickens and reported that melanin was present in all tissue layers of most organs, whereas in some tissue samples, melanin can only be observed in specific layers; however, the liver was the only organ that did not have melanin deposition, which coincides with the results of this study.

Melanin detection methods are commonly based on the chemical property that melanin is insoluble in acidic or neutral solvents [20,21]. In this study, the results revealed that the mouth skin of Liancheng white duck had the highest pigment value, and the skin and muscle tissues had the lowest values. Notably, the liver showed the second-highest pigment value in the extraction-based assay, whereas obvious melanin granules were not readily observed in the HE and TB-stained sections. This discrepancy suggests that the liver result should be interpreted as a method-dependent pigment signal rather than direct histological evidence of abundant melanin deposition.

The acid-hydrolysis-based extraction method used in this study reflects the amount of acid-resistant pigment residue, but it is not completely specific for melanin. In liver tissue, other endogenous pigments, pigment-like polymers, or acid-resistant residues may be co-extracted and contribute to the measured value. Because the liver is a metabolically active organ involved in heme turnover, bile pigment metabolism, oxidation-related pigment accumulation, and phagocytic clearance, the measured acid-resistant residue may include heme- or bile-related pigment residues, lipofuscin-like oxidized materials, hemosiderin-related components, or other pigment-containing phagocytic cell products, rather than typical melanocyte-derived melanin granules alone. In addition, histological observation is field-dependent and may miss sparse or unevenly distributed pigment-containing cells. Chemical extraction reflects the total pigment-related residue from the sampled tissue, whereas routine HE and TB staining depend on the selected microscopic fields and the visibility of discrete pigment granules. Thus, a relatively high extraction-based value may occur even when typical melanin granules are not readily visible histologically, and this value does not necessarily indicate abundant typical melanin granule deposition in the liver.

Previous studies provide some biological support for the possible presence of melanogenesis-related components in avian liver. For example, Ye et al. [22] reported positive immunoreactivity of TYRP1 in liver parenchymal cells of Guyuan black-bone chickens, implying that melanogenesis-related components or melanin-associated pigments may exist in the liver in certain pigmented poultry. Moreover, studies in other vertebrates have described hepatic pigmentary systems in which pigmented macrophage-like cells contain melanin-related pigments and exhibit melanogenic enzyme activity, supporting the possibility that hepatic phagocytic cells may contribute to pigment accumulation in the liver [23,24,25]. This interpretation is also supported by reports showing that melanomacrophage centers are aggregates of pigmented phagocytes that may occur in the liver of many vertebrates, and that melanomacrophages can contain mixed pigments, including melanin, lipofuscin, and hemosiderin [26]. In teleost liver and hematopoietic tissues, melano-macrophages have also been reported to be melanin- and hemosiderin-positive and to participate in the phagocytic handling of pigment-containing cellular debris [27]. Therefore, the liver signal observed in this study may reflect a mixture of pigment-related residues or pigment-containing phagocytic cells rather than the uniform deposition of typical melanin granules.

Nevertheless, because our histological results did not show obvious melanin granule deposition in the liver, the extraction-based liver value should be regarded as a pigment-related signal rather than definitive evidence of melanin deposition. Further validation using melanin-specific methods, such as Fontana–Masson staining combined with melanin bleaching, Prussian blue staining to exclude hemosiderin, immunohistochemical detection of melanogenesis-related proteins such as TYR, TYRP1, or DCT, and chemical quantification of melanin-specific degradation products by HPLC or LC-MS, is required to confirm the exact nature of the pigment detected in liver tissue. Fontana–Masson staining can help identify melanin or argentaffin pigments, while bleaching controls can further support whether the signal is melanin-related. In addition, HPLC- or LC-MS-based detection of melanin-specific degradation products would provide stronger chemical evidence than acid-resistant residue measurement alone.

### 4.2. Whole-Transcriptome Analysis to Identify Candidate Regulatory Signals Associated with Melanin Deposition in Liancheng White Ducks

Whole-transcriptome profiling has been widely applied to identify candidate regulators of pigmentation, such as EDN3–ncRNA interactions in black-bone chickens [28], miRNA–TYRP1 regulation in fragrant pigs [29], and transcriptome–metabolome evidence related to melanogenesis and tyrosine metabolism in sheep [30].

In our study, KEGG pathway enrichment analysis showed that DEGs were mainly associated with tyrosine metabolism, melanogenesis, and the MAPK and Wnt signaling pathways, which have also been linked to pigmentation regulation in other animal models [31,32,33,34,35]. These results suggest that tissue-specific melanin deposition in Liancheng white ducks may involve multiple melanogenesis-related pathways.

In this study, MITF, TYR, TYRP1, DCT, WNT4, WNT7B, MC1R, KIT, and EDNRB were upregulated, whereas ASIP, ADCY2, and ADCY8 were downregulated in mouth skin. MITF is a central transcription factor essential for melanocyte development and regulates key melanogenic enzymes TYR, TYRP1, and DCT [36]. Therefore, the upregulation of these genes suggests activation of an MITF–TYR/TYRP1/DCT-centered melanogenic program in highly pigmented mouth skin [37].

In addition, the upregulation of WNT4 and WNT7B indicates that Wnt-related signaling may participate in melanogenesis regulation [38]. MC1R, KIT, and EDNRB are involved in melanin synthesis, melanocyte survival, migration, differentiation, and feather-color formation [39,40,41,42,43], suggesting that pigmentation differences may result from coordinated changes in melanocyte development and melanogenic signaling.

Conversely, ASIP competitively inhibits α-MSH signaling, reducing eumelanin production by blocking MC1R. Its association with pigmentation traits has been reported in cattle and geese [44,45]. ADCY family members regulate cAMP signaling and have been implicated in melanogenesis and pigment dispersion [46,47,48]. In this study, the downregulation of ADCY2 and ADCY8 may represent an isoform-specific regulatory signal associated with pigmentation; however, their direct roles in melanin production require further functional validation.

### 4.3. Construction of a ceRNA Regulatory Network to Explore Potential Regulatory Associations Related to Melanin Deposition in Liancheng White Ducks

Noncoding RNAs (ncRNAs) play important regulatory roles in many biological processes [49,50]. In this study, 18 DEmiRNAs and 364 DElncRNAs were screened from the mouth skin and skin of Liancheng white ducks, and the KEGG enrichment analysis on the target genes revealed that the target genes of the DEmiRNAs (novel_18, novel_290, apl-miR-11588-3p and novel_361) were enriched mainly in herpes simplex virus 1 infection pathway, the MAPK signaling pathway, and the focal adhesion pathway; the target genes (TYR, TYRP1, CBR1, HPGDS and GPX2) of the DElncRNAs were enriched in the metabolic pathways and the arachidonic acid metabolism pathway.

The herpes simplex virus 1 infection pathway involves the target genes PI3K and JAK1. PI3K/Akt/GSK3β and JAK related signaling pathways have been reported to affect MITF activity, tyrosinase activity, and melanin production [51,52,53,54,55,56]. Therefore, novel_290 and novel_18, which were predicted to be linked to PI3K and JAK1, may represent candidate miRNAs associated with melanogenesis-related signaling modulation, although functional validation is still required.

Similarly, the MAPK signaling pathway included MAPK8IP3 (JIP3), a scaffold protein participating in JNK and MAPK signaling. Prior work has connected MAPK-related targets and pathways with pigmentation disorders or melanogenesis-related processes [57,58,59]. Moreover, miR-370-5p inhibited pigmentation and cell proliferation by downregulating the expression of MAPK8 in sheep melanocytes [60]. Therefore, apl-miR-11588-3p, which was predicted to target MAPK8IP3 in our study, may serve as a potential regulatory candidate linking ncRNA regulation to MAPK-associated melanogenesis control.

The focal adhesion pathway included ILK, which is involved in cytoskeletal remodeling, melanosome transport, melanocyte migration, and dendrite formation [61,62,63,64]. The DEmiRNA novel_361, which was predicted to correspond to ILK, may be associated with melanin transfer and transport-related cellular processes.

Based on the KEGG pathway enrichment analysis results, DElncRNAs may be associated with these pathways through their predicted target genes, thereby potentially participating in the process of melanogenesis. The metabolic pathways included the target genes TYR and TYRP1, and their corresponding DElncRNAs may be involved in tissue melanin production; the arachidonic acid metabolism pathway included the target genes CBR1, HPGDS, and GPX2. Because arachidonic acid metabolism, HPGDS, and GPX family members have been associated with pigmentation- or melanogenesis-related processes in previous studies [65,66,67], DElncRNAs corresponding to CBR1, HPGDS, and GPX2 may represent candidate ncRNAs associated with pigment-related metabolic regulation.

The ceRNA network was analyzed, and the relationship pairs were identified and functionally annotated. Among these candidates, the ENSAPLT00000025522–apl-miR-11588-3p–MAPK8IP3 axis was identified in the MAPK signaling pathway. Because apl-miR-11588-3p was significantly downregulated in mouth skin, this axis may represent a candidate ceRNA regulatory association related to MAPK associated melanogenesis. It should be noted that ceRNA regulation requires several conditions, including direct miRNA binding, appropriate subcellular localization, sufficient expression abundance, and reciprocal regulation among lncRNAs, miRNAs, and mRNAs. In the present study, these conditions were not experimentally tested. Therefore, the predicted ENSAPLT00000025522–apl-miR-11588-3p–MAPK8IP3 relationship should be interpreted as a candidate regulatory association rather than a confirmed ceRNA mechanism. Future studies should verify this relationship using dual-luciferase reporter assays to confirm miRNA binding, knockdown, or overexpression assays to test regulatory direction, and rescue experiments to determine whether the lncRNA–miRNA–mRNA interaction affects melanogenesis-related phenotypes.

One further limitation of this study is that pooled samples were used for transcriptomic sequencing. Each RNA-Seq library was generated from equal amounts of the same tissue collected from five ducks within one biological replicate group, yielding four pooled biological replicates per tissue type. Although this design reduced random individual variation and enabled the identification of major tissue-level expression differences, it limited the assessment of inter-individual variability in gene expression. Accordingly, the DEGs, DEmiRNAs, DElncRNAs, and predicted ceRNA relationships identified in this study should be regarded as candidate tissue-level regulatory signals. Future studies based on non-pooled individual samples and larger sample sizes are required to further validate these findings and clarify individual-level regulatory variation.

## 5. Conclusions

This study suggests that melanin in Liancheng white ducks is primarily distributed in the basal layer of the epidermis and around feather follicles, with tissue pigment values varying among mouth skin, liver, foot skin, muscle, and skin. The relatively high liver value observed in the extraction-based assay should be regarded as a method-dependent acid-resistant pigment-related signal rather than as direct evidence of abundant melanin deposition. Further melanin-specific histochemical and chemical validation is required to determine the nature of this liver pigment signal. Transcriptome analysis identified key genes (e.g., MITF, TYR, TYRP1, DCT, MC1R, KIT, EDNRB) and regulatory non-coding RNAs (e.g., novel_290, apl-miR-11588-3p, TCONS_00063335) potentially associated with tissue-specific pigmentation and melanogenesis-related pathways. The interaction pair ENSAPLT00000025522–apl-miR-11588-3p–MAPK8IP3 was proposed as a candidate ceRNA regulatory association that may be involved in this process. These findings provide candidate molecular information for the further investigation of tissue-specific pigmentation in Liancheng white ducks and generate testable hypotheses regarding melanogenesis-related regulatory networks. However, the predicted ENSAPLT00000025522–apl-miR-11588-3p–MAPK8IP3 relationship was inferred mainly from bioinformatic prediction, expression association, and pathway annotation. Further functional validation, including miRNA-target binding assays, lncRNA or miRNA knockdown or overexpression, rescue experiments, and phenotypic validation of melanin production, is required to determine whether these predicted associations represent functional ceRNA-mediated regulatory mechanisms.

## Figures and Tables

**Table 1:** KEGG enrichment list for the DEGs (partial).

KEGG ID	Description	GeneRatio ^1^	BgRatio ^2^	*p*-Value
apla04142	Lysosome	50/896	106/4341	5.44 × 10^−10^
apla04512	ECM–receptor interaction	35/896	77/4341	6.92 × 10^−7^
apla04510	Focal adhesion	62/896	172/4341	1.35 × 10^−6^
apla04020	Calcium signaling pathway	67/896	193/4341	2.37 × 10^−6^
apla04145	Phagosome	37/896	102/4341	0.000163
apla00360	Phenylalanine metabolism	9/896	16/4341	0.001829
apla00590	Arachidonic acid metabolism	18/896	46/4341	0.003023
apla00350	Tyrosine metabolism	12/896	29/4341	0.008777
apla04010	MAPK signaling pathway	62/896	228/4341	0.009005
apla04310	Wnt signaling pathway	39/896	133/4341	0.01003
apla04916	Melanogenesis	24/896	76/4341	0.015822

^1^ GeneRatio refers to the proportion of differential genes annotated to a specific pathway. It is calculated as the number of DEGs in the pathway divided by the total number of DEGs, and is used to assess the degree of enrichment relative to the background (BgRatio). ^2^ BgRatio refers to the proportion of genes in the background gene set annotated to a specific KEGG pathway. It is calculated as the number of background genes in the pathway divided by the total number of genes in the background set, typically including all detected genes.

## Data Availability

The datasets generated and/or analyzed during the current study are available in the GSA repository (GSA, https://ngdc.cncb.ac.cn/gsa/, accessed on 25 March 2025), the assigned accession of the submission is: CRA023941 (https://bigd.big.ac.cn/gsa/browse/CRA023941, accessed on 25 March 2025).

## Supplementary Materials

The following supporting information can be downloaded at: https://www.mdpi.com/article/10.3390/ani16121891/s1, Figure S1: KEGG functional enrichment of ceRNAs; Table S1: Diet composition and nutrient levels (air-dried basis); Table S2: Primer sequences; Table S3: miRNA primer sequences (by Tailing A).

## Abbreviations

The following abbreviations are used in this manuscript:

DEGs	Differentially expressed genes
DEmiRNA	Differentially expressed microRNA
DElncRNA	Differentially expressed long noncoding RNA
ceRNA	Competitive endogenous RNA
qRT-PCR	Quantitative reverse transcription-polymerase chain reaction
MITF	Melanocyte inducing transcription factor
TYR	Tyrosinase
ASIP	Agouti signaling protein
ADCY2	Adenylate cyclase 2
PI3K	Phosphatidylinositol 3-kinase
JAK1	Janus kinase 1
TYRP1	Tyrosinase-related protein 1
DCT	Dopachrome tautomerase
MC1R	Melanocortin 1 receptor
EDN3	Endothelin 3
SNP	Single nucleotide polymorphism
GSH	Glutathione
MREs	microRNA response elements
TB	Toluidine blue
GO	Gene Ontology
KEGG	Kyoto Encyclopedia of Genes and Genomes
GAPDH	Glyceraldehyde-3-phosphate dehydrogenase
FPKM	The fragments per kilobase of transcript per million fragments mapped
WNT4	Wnt family member 4
WNT7B	Wnt family member 7B
KIT	Receptor tyrosine kinase
EDNRB	Endothelin receptor type B
ADCY8	Adenylate cyclase 8
MAPK8IP3	Mitogen-activated protein kinase 8 interacting protein 3
ILK	Integrin linked kinase
CBR1	Carbonyl reductase 1
HPGDS	Hematopoietic prostaglandin D synthase
GPX2	Glutathione peroxidase 2
VANGL1	VANGL planar cell polarity protein 1
SEC24B	SEC24 homolog B
DOPA	L-3,4-Dihydroxyphenylalanine
GSK3β	Glycogen synthase kinase 3 beta
JIP3	JNK-interacting protein 3
JNK	c-Jun N-terminal kinase
FDR	False discovery rate
