# Supplementary material for: Melanin Deposition and Screening of Melanogenesis-Related Differential RNAs and Construction of ceRNA Regulatory Network in Liancheng White Ducks
Source: Animals (Basel). 2026 Jun 18;16(12):1891. doi: 10.3390/ani16121891 (PMC13295401; doi:10.3390/ani16121891)
Supplement: Supplementary file 1 [file animals-16-01891-s001.zip › Supplementary Table S1..pdf]

Table S1. Diet composition and nutrient levels (air-dried basis).

Item	Content	Item	Content
Diet composition		Nutrient levels	
Corn/%	50.5	Crude protein/%	16.95
Wheat bran/%	8.2	Crude fiber/%	3.47
Soybean meal/%	26.6	Crude ash/%	11.88
Calcium hydrogen phosphate/%	0.9	Calcium/%	3.30
Limestone powder/%	8.5	Available phosphorus/%	0.69
Salt/%	0.3	Methionine/%	0.34
Premix/%	5.0	Lysine/%	0.90
		Metabolizable energy/MJ·kg^{-1}	10.9

Note: The premix provided the following nutrients per kg of diet: vitamin A, 12,000 – 19,000 IU; vitamin D_3, 2,000 – 3,000 IU; vitamin E, 20 mg; vitamin K_3, 15 mg; vitamin B_1, 3.0 mg; vitamin B_2, 3.5 mg; vitamin B_6, 8.0 mg; D-pantothenic acid, 28.5 mg; niacin, 25 mg; folic acid, 0.6 mg; iron, 100 mg; manganese, 90 mg; zinc, 90 mg; iodine, 0.50 mg; and copper, 10 mg. Metabolizable energy was a calculated value, while the other values were measured values.
