# Supplementary material for: Melanin Deposition and Screening of Melanogenesis-Related Differential RNAs and Construction of ceRNA Regulatory Network in Liancheng White Ducks
Source: Animals (Basel). 2026 Jun 18;16(12):1891. doi: 10.3390/ani16121891 (PMC13295401; doi:10.3390/ani16121891)
Supplement: Supplementary file 1 [file animals-16-01891-s001.zip › Supplementary Table S2..pdf]

Table S2. Primer sequences.

Gene	GenBank Accession	Primer Sequence (5' to 3')	Size , bp
GAPDH	XM_005016745.2	F:GGAGCTGCCCAGAACATTATC<br>R:GCAGGTCAGGTCCACGACA	141
ADCY2	XM_038175758.1	F:CTTGGAATGTCTCAGGCTGCTAA<br>R:CATGTAAGTGCTCCCAATGGTCT	112
ASIP	XM_038166012.1	F:GTCCACCCAAATCCGCATCT<br>R:GCAGGGAAGGCAGGAAACT	108
MC1R	NM_001310805.1	F:CAGCGAGGGCAACCAGAG<br>R:CCAGCGTGAGGAAGAGTTCGT	92
TYR	XM_005029583.5	F:GATGAACAATGGCTCCAACCTC<br>R:CACATTGGACCCACCTAAGAGT	109
