# Supplementary material for: Melanin Deposition and Screening of Melanogenesis-Related Differential RNAs and Construction of ceRNA Regulatory Network in Liancheng White Ducks
Source: Animals (Basel). 2026 Jun 18;16(12):1891. doi: 10.3390/ani16121891 (PMC13295401; doi:10.3390/ani16121891)
Supplement: Supplementary file 1 [file animals-16-01891-s001.zip › Supplementary Table S3..pdf]

Table S3. miRNA primer sequences (by Tailing A).

Gene	Forward Primer (5' to 3')	Annealing, ℃
U6-F	CTCGCTTCGGCAGCACA	60
novel 18-F	CGCGTGGACGGAGAACTGAT	60
novel 120-F	GCGTCAGCACGTGTTCAGTTT	60
novel 346-F	GCTGAACTCCCCCAGGAC	60
novel 361-F	CGCGCCTGTGCCAGCCCT	60
universal reverse primers	provided by the miRNA 1st Strand cDNA Synthesis Kit (by Tailing A)	
