# Supplementary figures and images for: Melanin Deposition and Screening of Melanogenesis-Related Differential RNAs and Construction of ceRNA Regulatory Network in Liancheng White Ducks
Source: Animals (Basel). 2026 Jun 18;16(12):1891. doi: 10.3390/ani16121891 (PMC13295401; doi:10.3390/ani16121891)

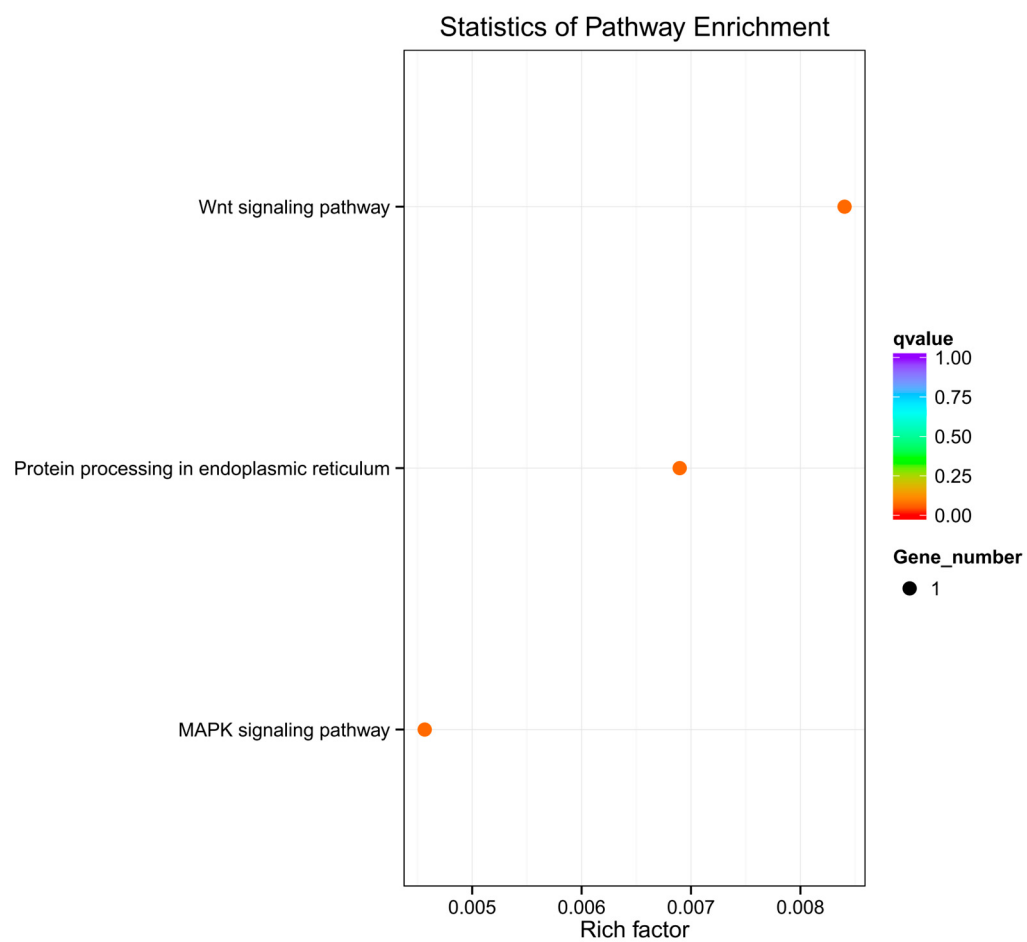

**Figure S1.** KEGG functional enrichment of ceRNAs.

Supplement: Supplementary file 1 [file animals-16-01891-s001.zip › Supplementary Figure S1.pdf]
